# Use of liquid biopsy in monitoring therapeutic resistance in *EGFR* oncogene addicted NSCLC

**DOI:** 10.37349/etat.2020.00024

**Published:** 2020-12-28

**Authors:** Marialucia Iacovino, Vincenza Ciaramella, Fernando Paragliola, Gabriella Suarato, Gesualdina Busiello, Francesca Sparano

**Affiliations:** Medical Oncology, Department of Precision Medicine, Università degli Studi della Campania Luigi Vanvitelli, 80131 Naples, Italy; University of Southampton, UK

**Keywords:** Liquid biopsy, non small cells lung cancer, osimertinib, EGFR resistance, T790M

## Abstract

Liquid biopsy has emerged as a minimally invasive alternative to tumor tissue analysis for the management of lung cancer patients, especially for epidermal growth factor receptor (*EGFR*) oncogene addicted tumor. In these patients, despite the clear benefits of tyrosine kinase inhibitors therapy, the development of acquired resistance and progressive disease is inevitable in most cases and liquid biopsy is important for molecular characterization at resistance and, being non-invasive, may be useful for disease monitoring. In this review, the authors will focus on the applications of liquid biopsy in *EGFR*-mutated non small cells lung cancer at diagnosis, during treatment and at progression, describing available data and possible future scenarios.

## Introduction

Liquid biopsy consists in the analysis of tumor circulating materials derived from body fluids. Since a plethora of anticancer drugs are currently accessible for lung cancer treatments, an iterative assessment of tumor’s biomarkers is the ideal approach to define the best sequence of therapies for each patient. In this scenario, liquid biopsy can be used as a non-invasive method to detect any targetable genetic mutations and to select a corresponding targeted therapy, to monitor response to treatment and to identify eventual genomic alterations occurred at resistance. Moreover, liquid biopsy provides information on the genomic profile of the whole tumor landscape, overcoming spatial and temporal heterogeneity [1].

From body fluids samples of metastatic cancer patients, the circulating free DNA (cfDNA) is usually found in small quantities. Only a small portion of cfDNA derives specifically from tumor cells, defined as circulating tumor DNA (ctDNA), and can be analyzed by next-generation sequencing (NGS) [2]. NGS is the standard method used to sequence in short time large genomic regions, allowing to detect several genetic alterations, such as single nucleotide variants, insertions and deletions, gene fusions and copy number variations with high sensitivity and specificity [3].

Liquid biopsy is extremely relevant in lung cancer patients, as the tumor is often difficult to reach with surgical or radiological biopsies, which are anyway invasive and potentially risky procedures. In fact, almost three-fourths of lung cancers patients with advanced disease are diagnosed via cytology samples because the acquisition of histologic specimens is not possible, resulting in a 30% of cases in which the sample is not sufficient for biomarker evaluation, either at diagnosis or at disease progression [4].

In oncogene-driven non small cells lung cancer (NSCLC) patients, NGS of ctDNA can be successfully utilized to select appropriate biomarker-oriented treatments [5]. For patients affected by metastatic NSCLC, activating epidermal growth factor receptor (*EGFR*) mutations occur in almost 10–12% of cases and tyrosine kinase inhibitors (TKIs) are the standard front-line therapy [6].

In this review we will discuss on current and possible future application of liquid biopsy in EGFR-driven NSCLC, defined as lung cancers harboring targetable mutation in *EGFR* genes.

## Clinical applications of liquid biopsy in *EGFR* oncogene addicted NSCLC

### Basal assessment for treatment-naïve patients

The assessment of predictive biomarkers is essential to select the best treatment for NSCLC patients. Thus, liquid biopsy, as stated, could provide clinical advantages in treatment-naive patient when tumor specimens are scarce and it could also spare diagnostic tissue for additional molecular investigations, including immunohistochemistry.

Therefore, it is especially recommended when tumor tissue is insufficient, unreachable, or the procedures to obtain tumor sample are expected to cause a potential delay longer than 2 weeks [7].

However, not all tumors shed sufficient amount of DNA into peripheral circulation for detection and the sensitivity of liquid biopsy in advanced disease is approximately 85%. A false negative result of plasma analysis is more frequent in treatment-naïve patients with indolent, slow-growing tumors. For this reason, a negative result must be interrogated further with a tumor biopsy [8].

### Evaluation after first line therapy of *EGFR*-mutant NSCLC

First-(gefitinib, erlotinib) and second-(afatinib, dacomitinib) generation EGFR TKIs have been considered the standard first-line therapy for many years for *EGFR* mutant NSCLC but, even though the responses are generally solid and sustained, the onset of acquired resistance is universal and occurs mostly through on-target mechanisms (EGFR-dependent mechanisms) after a median of 9 to 12 months of treatment [9–11].

It is highly recommended for patients experiencing progressive disease (PD) during TKIs treatment to repeat a tissue biopsy at the progressing site to evaluate any potential mechanisms of resistance. However, the use of ctDNA extracted from plasma samples may be used to identify mechanisms of resistance to EGFR-TKIs, thus overcoming all the several technical limitations of re-biopsy and avoiding invasive procedures.

In approximately 50% of patients, resistance to first generation EGFR-TKI gefitinib or erlotinib is due to the development of the secondary T790M mutation in exon 20. Osimertinib is a third-generation EGFR-TKI designed to target T790M positive (T790M+) EGFR in patients who have acquired this mechanism of resistance to earlier generation TKIs (phase III AURA3 trial: AZD9291 *versus* platinum-based doublet-chemotherapy in locally advanced or metastatic non-small cell lung cancer, NCT02151981) [6, 12].

After publication the results from the FLAURA trial, that compared osimertinib to first generation TKIs in untreated *EGFR* mutant NSCLC, osimertinib has been approved also in the first-line setting, representing, to date, the treatment of choice in this setting [13].

Some data of mechanisms of resistance to first line osimertinib are already available as is now the standard of care for *EGFR* mutant [both T790M+ and T790M negative (T790M-)] NSCLC, including genomic alterations that are detectable by liquid biopsy as described below.

Otherwise, in patients with T790M-NSCLC a tumor re-biopsy is recommended to define the tissue T790M status and, if confirmed as negative, platinum doublet chemotherapy is the standard second-line treatment [14].

T790M-NSCLC patients represent a heterogeneous group and analysis of plasma ctDNA may show a diversified genetic landscape. The most frequent off-target resistance mechanisms include bypass pathway activation, such as *MET* amplification (estimated to occur in 5% to 20% of patients) or *HER2* amplification (in up to 8% of patients), activation of other pathways like AXL, Hedgehog and downstream signals (e.g., *PI3K* and *BRAF* mutations) [15–18]. Other known mechanisms of resistance are histological transformations in small cell lung cancer (SCLC) and epithelial-to-mesenchymal transformation [19].

### Liquid biopsy after second line therapy of *EGFR* mutant NSCLC with osimertinib

ctDNA analysis has an important role not only to identify T790M+ patients after progression to first- and second-generation EGFR TKIs and before starting osimertinib, but also during second line treatment to monitor disease evolution, predict efficacy outcomes and even at the time of progression to determine resistance mechanisms and to establish adequate combination therapies [20].

**Figure 1.**
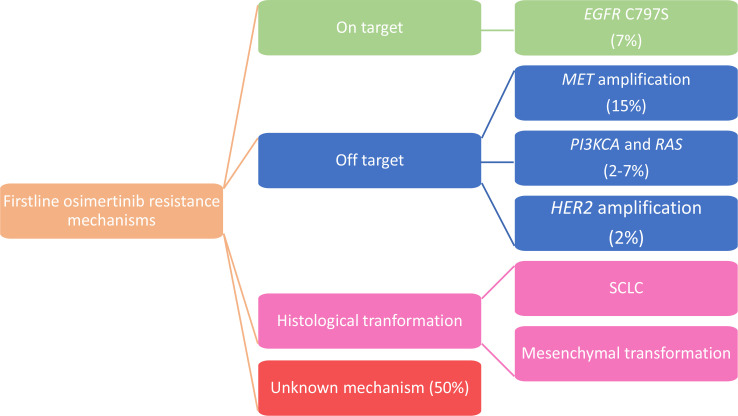
Mechanisms of acquired resistance to second-line osimertinib

In the AURA3 trial an analysis of the ctDNA genomic profile was performed in T790M+ patients who experienced PD during osimertinib treatment. Among the resistance mechanisms detected, *MET* amplification (19%) and *EGFR* C797S (15%) were the most frequent ones. It was also estimated that 49% of patients lost T790M mutation at the time of PD and in those cases resistance to second-line osimertinib is often associated with the development of competing resistance mechanisms such as *KRAS* mutations, *MET* amplification, SCLC transformation and gene fusion [21] (Figure 1).

Moreover, an early resistance to osimertinib and a shorter time to progression (6.1 *vs.* 15.2 months) were related to the loss of the T790M mutation, probably connected to the prevalence of resistant clones already present before the beginning of treatment. Serial T790M status profiling could be used as an intuitive method to identify these two biologically different types of osimertinib resistance [22].

Bordi et al. [23], also explored the role of ctDNA testing to monitor second line osimertinib therapy revealing a poor reliability of T790M for prognostic purpose and an important role in prognostic stratification for all *EGFR* activating mutation, since patients with absence or low levels of mutations presented a better outcome to osimertinib. Thus, further studies are still needed in this context. Among the other resistance mechanisms detected in the AURA3 ctDNA analysis beyond *MET* amplification (19%) and *EGFR* C797S tertiary mutation (15%), amplifications in *HER2* (5%), and *PIK3CA* (4%), mutations in *BRAF* V600E (4%) and *KRAS* (1%), and oncogenic fusions in *FGFR3*, *RET* and *NTRK* (4%) were described [21].

The *EGFR* C797S is a tertiary mutation at the drug’s binding site on the EGFR receptor and has been demonstrated to be an acquired mechanism of resistance to osimertinib in ~20–30% of cases by NGS of ctDNA, commonly detected along with other resistance mechanisms in ctDNA. When the mutations are in *cis*, the cells were resistant to all available EGFR-TKIs alone as well as combined. These findings are leading to the development of 4th generation TKIs with activity against C797S mutations that is still in progress [24].

Multiple other tertiary resistance *EGFR* mutation such as G724, L792, L718 and G719, have been detected using targeted NGS, mainly on cfDNAs [25].

In comparison with the first and second generation TKIs, the resistance mechanisms observed is mostly off-target, underlining a more effective on-target inhibition mediated by osimertinib and differences in selective pressure and clonal evolution as compared to previous generations of TKIs [26].

*MET* gene amplification represents the most frequent cause of bypass pathway activation resistance mechanism to EGFR-TKIs, leading to osimertinib resistance by persistent activation of signaling pathways downstream of EGFR. In order to overcome resistance, multiple agents have been studied to target MET or its ligand hepatocyte growth factor (HGF) and several MET inhibitors have been studied in association with EGFR TKIs or chemotherapeutic agents in NSCLC patients who developed resistance to TKIs [27].

*HER2* amplification has been determined in 5% of patients with acquired resistance to second-line osimertinib, and can coexist with *EGFR* L792X + C797X + *PIK3CA* amplification (in 1% of cases), *EGFR* G796S + *MET* amplification (1%), and *PIK3CA* amplification alone (1%) [28]. A phase I/II trial to evaluate TAK-788, an experimental drug targeting *EGFR* and *HER2* mutations, including exon 20 insertions, is currently ongoing (NCT04129502) [29].

Moreover, other data are available from preclinical studies. The combination of osimertinib and trastuzumab-emtansine (T-DM1) has been shown to overcome *HER2* amplification-mediated resistance in EGFR-T790M+ NSCLC cell lines [30]. Also, our group has demonstrated that epithelial-to-mesenchymal transition (EMT) and activation of AXL, Hedgehog and MAPK pathway can co-occur in murine xenografts of *EGFR*-mutant NSCLC treated with a sequence of first-generation EGFR-TKI, second-generation EGFR-TKI afatinib and osimertinib [31].

Hsu et al. [32], recently presented a clinical case of an exon 16 skipping *HER2* deletion causing resistance to osimertinib in an EGFR-T790M+ patient. *HER2* exon 16 skipping mutation has only previously been described in breast cancer, in which it is reported as an oncoprotein that activates Src kinase signaling. The mutated tumor cells resulted to be resistant to Src inhibition with or without osimertinib, but the combination therapy with osimertinib and the pan-HER2 TKI afatinib may be effective. As the mutation was already present before treatment, this case highlights the relevance of obtaining a genomic characterization to determine preexisting alterations which may have both prognostic and predictive value for therapeutic implications; similarly, this study also demonstrates that repeating molecular analyses at multiple time points is useful to discover new potentially actionable molecular targets [33].

Another mechanism of resistance to late generation TKIs treatment is reported to be the transformation to SCLC, known to be difficult to assess through liquid biopsy [34]. Neverthless, Tsui et al. [20], demonstrated that ctDNA may be analyzed in terms of global copy number variations to evaluate its dynamism in patients with a histological transformation into SCLC. In fact, analysis of plasma samples, collected after transformation, has shown copy number alterations associated with SCLC such as *MYCL1*, *SOX2*, and *SOX4*. In addition, *TP53* mutations were found to be at low levels before EGFR-TKI initiation in patients baseline plasma samples. Being *TP53* mutation universally present in all SCLC, the pre-existing clone of *TP53* mutant cells may growth selectively under the pressure of EGFR-TKIs treatment and could explain the SCLC-like resistance mechanism. The levels of *TP53* mutant cells in plasma were also higher with disease progression and lower when patient experienced clinical response during SCLC-directed chemotherapy [35, 36].

### Liquid biopsy after first line therapy of *EGFR* mutant NSCLC with osimertinib

**Figure 2.**
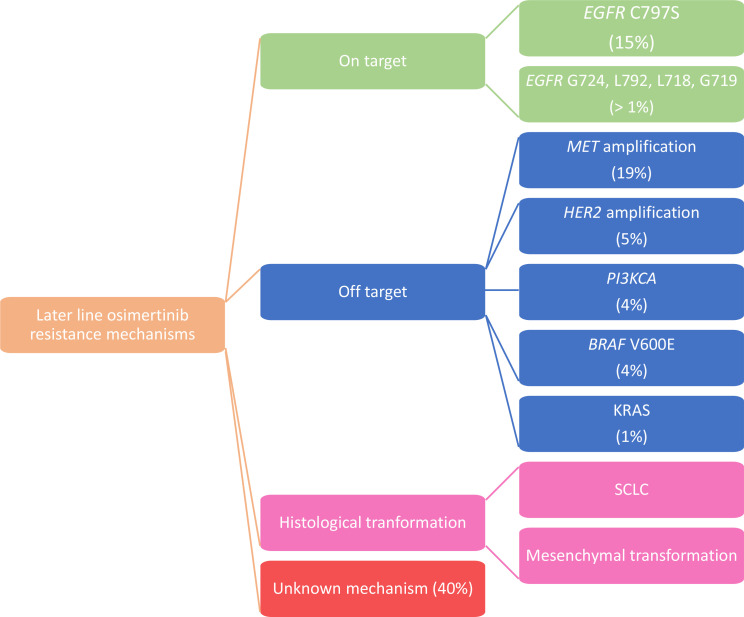
Mechanisms of acquired resistance to first-line osimertinib

As in the AURA3 trial, in the phase III FLAURA study, paired plasma samples were collected at baseline and at PD and/or treatment discontinuation and were analyzed using NGS to determine alteration that may have caused acquired resistance to first-line osimertinib. Preliminary data revealed that in the osimertinib arm, there was no evidence of acquired EGFR T790M, as expected knowing the specificity of osimertinib for T790M, and the most frequent acquired resistance mechanism detected was *MET* amplification (15%), followed by *EGFR* C797S mutation (7%); other mechanisms described were *HER2* amplification (2%), *PIK3CA* and *RAS* mutations (2–7%) [37] (Figure 2).

From these data, we can notice that some of the mechanisms are common between osimertinib administered as a first- and second-line therapy but resistance to second line osimertinib is more reliant on on-target alterations with a much higher frequency of C797S alterations compared to resistance to front line osimertinib.

In particular, when osimertinib was given as a first-line therapy, *MET* amplification was detected in 15% of patients by NGS ctDNA analysis, being the prevalent resistance mechanism. It is noteworthy that gene amplification is probably underreported in plasma compared to tissue analysis.

Mechanisms of resistance and their detection are still under study and new treatments for patients after progression to first-line osimertinib are urgently required. In view of what we reported up to now, the results of the following two studies will be relevant.

The ELIOS study (NCT03239340) is a phase II, open-label, single-arm trial designed to evaluate the efficacy, safety and resistance mechanisms to osimertinib when administered as first-line therapy in patients with locally advanced or metastatic *EGFR*-mutated NSCLC. In this trial, plasma genotyping together with paired tumor biopsy will be analyzed by NGS, providing also more information about concordance between the two approaches.

We are also expecting the results of the phase II ORCHARD trial (NCT03944772) that aims to investigate molecular-guided treatment options as second line after progression on osimertinib by evaluating potential targetable acquired resistance mechanisms. Patients will receive study treatment according to the mutation encountered: osimertinib plus gefitinib, osimertinib plus necitumumab,osimertinib plus savolitinib, platinum-based doublet plus durvalumab.

### Current status and future perspectives of liquid biopsy

As stated above, liquid biopsy can be utilized to dynamically detect resistance mutations in course of treatment with EGFR inhibitors and, when the tumor tissue biopsy is not possible, ctDNA analysis is the only available method for the identification of patients that can benefit from a TKI therapy.

However, an important limitation is the unsuccessful concordance between the mutational status of cfDNA and tumor DNA, maybe due to the low quantity of ctDNA that makes difficult the evaluation of the heterogeneity of tumor mutational landscape. To overcome this issue, other body fluids in sites adjacent to metastases have been studied as an alternative and more reliable source of ctDNA. In fact, higher levels of cfDNA were extracted from other body fluids such as cerebrospinal fluid, pleural effusion, and ascites, resulting in a valuable method for detecting tumor biomarkers [38].

In addition to the cfDNA, circulating tumor cells (CTCs), miRNAs and exosomes are other tumor-derived material that could be reliable surrogates to investigate tumor biology. In a pre-clinical setting these evaluations revealed promising results for their possible application in screening programs or as prognostic/predictive biomarkers and their applications will probably shift to the clinical scenario in the near future.

CTCs reflect tumor properties providing genomic, transcriptomic, and proteomic information on the tumor and, in addiction, may also be used to create preclinical models such as organoid cultures and murine models [39–40]. Tumor derived exosomes present specific surface proteins that reflect the tumor cell of origin and for this reason can be utilized to determine the mutational profile of tumor cells. miRNAs are a class of non-coding RNA molecules that play a role in post transcriptional regulation of gene expression. The analysis of multiple miRNAs expression levels has been proven to be potentially useful for early diagnosis, prognostic, and predictive purpose in a variety of tumors. However, these assessments are still affected by two important limitations: the lack of standard evaluations methods and the sample contamination by other cell components [41].

## Conclusion

Liquid biopsy already implemented clinical management of patients at diagnosis, during treatment and at progression offering a biomarker-oriented therapeutic option. Before starting treatment, liquid biopsy can be essential to investigate not only target mutations but also secondary mutations that can lead to resistance by clonal selection. During treatment, repeating serial liquid biopsies could have a role in monitoring disease evolution and anticipating a potential progression. At progression, liquid biopsy may identify mechanisms of resistance to select an appropriate treatment according to the therapy-dependent clonal selection, considering also that in the later-line osimertinib study, 36% of patients developed resistance through a targetable oncogene alteration. Nevertheless, no clear mechanism of resistance is established in approximately 40% of patients treated with second line osimertinib and in 50% of patients treated in first-line setting. Therefore, furthers trials to identify these unknown mechanisms are needed.

## Abbreviations

cfDNA: circulating free DNA
CTCs: circulating tumor cells
ctDNA: circulating tumor DNA
EGFR: epidermal growth factor receptor
HGF: hepatocyte growth factor
NGS: next-generation sequencing
NSCLC: non small cells lung cancer
PD: progressive disease
SCLC: small cell lung cancer
T790M-: T790M negative
T790M+: T790M positive
TKIs: tyrosine kinase inhibitors

## Declarations

### Copyright

© The Author(s) 2020.
